# Cardiovascular risk analysis in atopic dermatitis patients^☆^

**DOI:** 10.1016/j.abd.2023.09.008

**Published:** 2024-05-20

**Authors:** Lucas Pires Ventura, Mario Cezar Pires, Adilson da Costa

**Affiliations:** a Medical Assistance Institute, Hospital do Servidor Público Estadual de São Paulo, São Paulo, SP, Brazil; b Department of Dermatology, Complexo Hospitalar Padre Bento de Guarulhos, São Paulo, SP, Brazil

*Dear Editor,*

Atopic Dermatitis (AD), also known as atopic eczema, is a chronic, continuous or recurrent, pruritic inflammatory dermatosis with acute, subacute, or chronic eczematous lesions in typical locations that may vary according to age, but with a predilection for flexor regions and commonly associated with xerosis cutis.^1^ Its etiology is multifactorial and involves genetic and environmental factors, abnormal skin barrier, among others. It is more prevalent in children (10%‒20%) but may persist into adulthood (1%‒7%) and affects men and women equally.^2^ Treatment may vary according to the stage of the disease, age group, and severity, ranging from the use of moisturizers, topical corticosteroids, and calcineurin inhibitors for mild and moderate cases, to the use of systemic drugs such as cyclosporine, methotrexate, azathioprine, immunobiological (dupilumab) and, more recently, Janus Kinase inhibitors (baricitinib and upadacitinib) in severe cases.^3^

Chronic inflammatory diseases, such as AD, have been inconsistently associated with Cardiovascular Disease (CVD) and Cerebrovascular Accident (CVA) or stroke in epidemiological studies.^4^ Psoriasis and rheumatoid arthritis have been shown to have increased Cardiovascular risk (CVR) compared to the general population.^5^

The increasing prevalence of AD in adults makes it imperative to investigate associated comorbidities. Assessment of CVR is necessary for adequate management of AD and adoption of preventive measures, and the Framingham Risk Score (FRS) is one of the most studied instruments for individuals in primary health care,^6^ making it of great importance in the correlation analysis of AD and CVR.

Between June 2020 and July 2021, we conducted a pilot, case-control study in the Hospital do Servidor Público Estadual de São Paulo (IAMSPE), São Paulo, Brazil, and the Complexo Hospitalar Padre Bento (CHPB), Guarulhos, Brazil. This study was approved by the IAMSPE’s Ethics Committee (report nº 4.909.476) in which 60 subjects were equally distributed into both groups, matched for gender distribution, mean age, and associated comorbidities. The distribution in relation to ethnicity occurred in accordance with the general population attending the study, to represent the prevalence reported in the literature on cardiovascular diseases in the Brazilian population. However, this prevalence may vary from other already-published studies since it is directly dependent on some study particularities, such as the country of continental-dimension countries and substantial intermarriage.^7^

The main objectives of this study were to evaluate CVR in adults aged 30 years and older with AD, compare them to control subjects, and correlate AD severity with CVR. Stratification of CVR, which estimates the probability of outcomes such as acute myocardial infarction and CVA in the next 10 years, was applied through the FRS calculator, which considers parameters such as gender, age, serum total and HDL cholesterol levels, hypertension, diabetes, and smoking. Participants in the case group were selected according to the diagnostic criteria for AD proposed by the United Kingdom Working Party,^8^ and disease severity was assessed by the Scoring Atopic Dermatitis (SCORAD) index, which evaluates the extent and intensity of lesions, as well as the presence of subjective symptoms such as pruritus and sleep loss.^9^ The prolonged use of systemic therapy for the management of AD symptoms in patients who did not obtain adequate control after topical treatment of lesions was also taken into account as a complementary criterion in the estimation of severity. Drugs considered for pooled analysis were cyclosporine, methotrexate, azathioprine, and dupilumab.

The results of the analyses showed that there was no significant difference in CVR between the groups studied (p = 0.941) and that the variables evaluated were statistically independent (Table 1). However, age showed a strong correlation with increased CVR in participants in the case group (p < 0.001), as did systolic (p = 0.001) and diastolic blood pressure (p = 0.028) (Table 2). We did not observe a direct relationship between AD severity and CVR when using SCORAD (p = 0.388) (Table 2 and Fig. 1).

**Table 1:** Comparison of parameters associated with increased cardiovascular risk between groups.

		Average	Median	Standard deviation	Q1	Q3	N	IC	p-value
**Age**	Case	44.7	41	13.3	34	52	30	4.8	0.615
	Control	46.1	43	13.1	34	55	30	4.7	
**SBP**	Case	120.3	120	11.4	113	126	30	4.1	0.285
	Control	117.0	118	13.2	110	123	30	4.7	
**DBP**	Case	76.2	78	7.8	70	80	30	2.8	0.441
	Control	78.5	80	10.4	71	80	30	3.7	
**Cholesterol**	Case	189.5	191	34.5	168	211	29	12.5	0.355
	Control	197.7	190	29.9	181	218	30	10.7	
**HDL**	Case	53.8	54	13.7	43	60	29	5.0	0.234
	Control	49.7	50	11.5	42	54	30	4.1	
**CV Risk%**	Case	6.52	4.6	7.66	1.7	7.8	30	2.74	0.941
	Control	6.08	3.3	6.27	2.1	8.1	30	2.24	

SBP, Systolic Blood Pressure; DBP, Diastolic Blood Pressure; HDL, High Density Lipoprotein Cholesterol; CV Risk, Cardiovascular Risk.

**Table 2:** Correlation of cardiovascular risk with the main parameters assessed in the case group.

	CV Risk	
	Corr (r)	p-value
**Age**	0.800	<0.001
**SBP**	0.580	0.001
**DBP**	0.401	0.028
**Cholesterol**	0.299	0.115
**HDL**	-0.307	0.105
**SCORAD**	0.164	0.388

SBP, Systolic Blood Pressure; DBP, Diastolic Blood Pressure; HDL, High Density Lipoprotein Cholesterol; SCORAD, Scoring Atopic Dermatitis.

**Figure 1:**
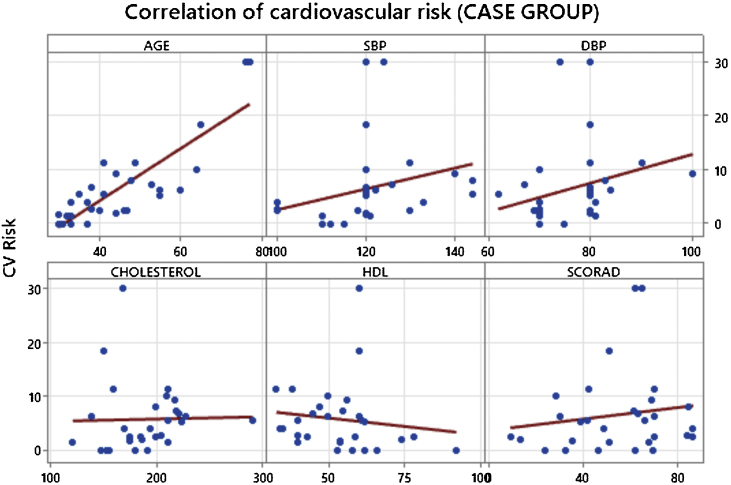
Correlation of cardiovascular risk with the main parameters assessed in the case group. SBP, Systolic Blood Pressure; DBP, Diastolic Blood Pressure; HDL, High Density Lipoprotein Cholesterol; SCORAD, Scoring Atopic Dermatitis.

When dividing the case group into severe and non-severe, adopting prolonged treatment with systemic medications as the severity criterion, and comparing them to the control group, we observed that there was no statistical difference in relation to CVR (p = 0.485) (Table 3).

**Table 3:** Comparison of severe, non-severe, and control cases in relation to CVR.

		Average	Median	Deviation standard	Q1	Q3	N	IC	p-value
**CV Risk in %**	Severe case	6.57	3	8.63	1	8	23	3.53	0.485
	Non-severe case	6.33	6	3.25	5	8	7	2.41	
	Control	6.05	3	6.30	2	8	30	2.25	

CV risk, Cardiovascular Risk; Severe case, Patients with severe atopic dermatitis classified by prolonged use of systemic therapy; Non-severe case, Patients with atopic dermatitis who do not use systemic therapy.

Recent studies in AD patients have shown divergent results regarding an increase in CVR. ^10^ In 2022, Ribeiro published a comprehensive review that evaluated a series of studies wherein the relationship between severe AD was evaluated regarding its inflammatory mechanisms and the risk of developing CVD.^10^ A total of 52 trials were included in that review, encompassing meta-analyses, cohorts, reviews, and cross-sectional studies. Thirty-six studies found a correlation between AD and CVD, while 16 did not. The authors concluded that there is insufficient evidence that AD alone is a risk factor for CVD and that CVR in moderate to severe AD may be a confounding variable.

Numerous risk-assessment instruments for the development of CV diseases have been created to date. We did not find any manuscript that evaluated CVR using the Framingham global risk score, which is one of the most studied CVR-stratification instruments and validated for use in the general population in primary health care. Using the Global Risk Score, we found that despite the fact that the case group had a higher average CVR than the control group, there was no statistically significant difference between them (p = 0.941). Using SCORAD to measure the severity of AD, we did not observe a significant correlation with CVR (p = 0.388). When classifying the severity of AD, taking into account the use of systemic medications, and comparing individuals with severe disease, non-severe disease, and the control group, we did not observe a statistically significant difference (p = 0.485), suggesting that the severity of AD does not directly imply the increased CVR.

Given the above, our primary findings corroborate the current guidelines of the American Academy of Dermatology,^11^ which, when evaluating a series of studies with a large number of participants with associated AD and increased CVR, did not identify evidence that demonstrated a magnitude of sufficient association to set a direct relationship up, concluding that screening for CVR in patients with AD would not be necessary. Even though there is a correlation between the endothelial inflammatory process and the presence of biomarkers associated with the Th2 response, and some evidence that demonstrates small associations of AD and elevated CVR, or some diseases that predispose it, there are currently no indications for further cardiovascular screening and treatment of patients with AD beyond what is already recommended for the general population.

We, therefore, conclude that although blood pressure and age are strongly related to elevated CVR in individuals in the case group, they are still not considered sufficient to claim that adult individuals with AD are more likely to develop CVD than individuals in the control group. The severity of AD, as measured by the SCORAD, as well as the use of systemic therapy, did not show a direct association with increased CVR, therefore, there is no need to perform additional screening measures for CVD in this population, and AD should therefore not be considered an independent factor for CVR. Therefore, we believe that CVR assessment for AD patients should be conducted according to age-based protocols and other associated factors.

## Financial support

None declared.

## Authors’ contributions

Lucas Pires Ventura: Substantial contributions to the conception or design of the work; acquisition and interpretation of data for the work; drafting the work; and agreement to be accountable for all aspects of the work ensuring that questions related to the accuracy or integrity of any part of the work are appropriately investigated and resolved.

Mario Cezar Pires: Analysis and interpretation of data for the work; final approval of the version to be published; and agreement to be accountable for all aspects of the work ensuring that questions related to the accuracy or integrity of any part of the work are appropriately investigated and resolved.

Adilson da Costa: Substantial contributions to the conception or design of the work; interpretation of data for the work; drafting the work and revising it critically for important intellectual content; final approval of the version to be published; agreement to be accountable for all aspects of the work in ensuring that questions related to the accuracy or integrity of any part of the work are appropriately investigated and resolved.

## Conflicts of interest

Lucas Pires Ventura: CAPES Scholarship.

Mario Cezar Pires: FQM (speaker), Pfizer (speaker and consultant), Sanofi (investigator), Lilly (investigator).

Adilson da Costa: None.
